# miR-96 exerts carcinogenic effect by activating AKT/GSK-3β/β-catenin signaling pathway through targeting inhibition of FOXO1 in hepatocellular carcinoma

**DOI:** 10.1186/s12935-019-0756-7

**Published:** 2019-02-20

**Authors:** Nanmu Yang, Jinxue Zhou, Qingjun Li, Feng Han, Zujiang Yu

**Affiliations:** 1 Department of Infectious Disease, The First Affiliated Hospital of Zhengzhou University, No. 1, Jianshe East Road, Zhengzhou, 450052 Henan China; 2 Department of Hepatopancreatobiliary Surgery, Henan Cancer Hospital, No. 127, Dongming Road, Zhengzhou, 450008 Henan China; 3 Department of Hepatopancreatobiliary Surgery, The Affiliated Tumor Hospital of Zhengzhou University, No. 127, Dongming Road, Zhengzhou, 450008 Henan China

**Keywords:** miR-96, FOXO1, HepG2 cells, AKT/GSK-3β/β-catenin, Proliferation

## Abstract

**Background:**

The aim of this research was to investigate the mechanism of miR-96 affecting hepatocellular carcinoma (HCC).

**Methods:**

mRNA and protein expression was detected by qRT-PCR and Western blot, respectively. HepG2 cells were transfected and grouped as follows: miR-NC group, miR-mimics group, NC + Vector group, mimics + Vector group, mimics + FOXO1 group. Luciferase reporter assay was performed. MTT and Transwell assay was conducted. In vivo studies by nude mice were performed. Immunohistochemistry and immunofluorescence was executed.

**Results:**

Up-regulated miR-96 and down-regulated FOXO1 was found in tumor tissues and HepG2 cells (*P *< 0.01). FOXO1 was directly suppressed by miR-96. Compared with NC + Vector group, mimics + Vector group has higher OD495 value (*P* < 0.05), higher migration and invasion cells (*P* < 0.01), larger transplanted tumor volume (*P* < 0.01), lower FOXO1 positive cell numbers (*P* < 0.01), higher p-AKT and p-GSK-3β expression (*P* < 0.01), lower p-β-catenin expression (*P* < 0.01), more β-catenin expression in the nucleus (*P* < 0.01). Compared with mimics + Vector group, mimics + FOXO1 group has lower OD495 value (*P* < 0.05), lower migration and invasion cells (P < 0.01), smaller transplanted tumor volume (*P* < 0.01), higher FOXO1 positive cells (*P* < 0.01), lower p-AKT and p-GSK-3β expression (*P* < 0.01), higher p-β-catenin expression (*P* < 0.01), less β-catenin expression in the nucleus (*P* < 0.01).

**Conclusion:**

miR-96 exerts carcinogenic effect by activating AKT/GSK-3β/β-catenin signaling pathway through targeting inhibition of FOXO1 in HCC.

## Background

Hepatocellular carcinoma (HCC) was one of the common malignant tumors, which posed a serious threat to the health and life of patients [1]. Over the past few decades, tremendous progress had been achieved in the treatment of HCC, such as liver transplantation [2]. However, HCC remained one of the major causes of cancer related deaths because of its high recurrence rate and metastatic rate [3]. In recent years, a large number of researchers were committed to exploring the molecular pathogenesis of HCC in order to find effective therapeutic targets for HCC.

miRNAs were a class of small non-coding RNAs that had been reported to regulate the expression of multiple tumor suppressor or oncogenes [4]. Accumulating evidence showed that miRNAs were involved in tumorigenesis and tumor progression [5]. HCC had also been found to be associated with aberrantly expression of a variety of miRNAs, including miR-96. miR-96 had been shown to be abnormally expressed in colorectal cancer, osteosarcoma, prostate cancer and several other malignant tumors [6–9]. A limited number of studies also showed obviously up-regulated miR-96 expression in HCC, and HCC progression was blocked by suppressing miR-96. For example, Wang et al. [10] noticed that miR-96 acted as onco-miRNAs in HCC. Suppression of miR-96 led to reduced HCC cell proliferation and migration. Baik et al. [11] confirmed the role of miR-96 in promoting tumor growth by implanting miR-96-overexpressing HepG2 cells in a xenograft model. Chen et al. [12] declared that high serum miR-96 level was remarkably related to higher prevalence of lymph node metastasis and higher overall survival rate in HCC patients. But unfortunately, the exact molecular mechanism by which miR-96 affected HCC progression had not been clearly identified.

FOXO1 was considered a tumor suppressor gene in HCC, which was reported to reverse epithelial-interstitial transformation by inhibiting invasion and metastasis of HCC cells [13]. Jiang et al. [14] researched that some drugs (such as Trifluoperazine) might inhibit HCC growth via activating FOXO1. In HCC, FOXO1 has also been found to be regulated by several miRNAs. Zeng et al. [15] revealed that miR-135a enhanced HCC cells migration and invasion via suppressing FOXO1. Lou and colleagues [16] illustrated that miR-142-5p could impair growth and stimulate apoptosis of HCC cells through enhancing FOXO1 expression. However, the mechanisms by which miR-96 and FOXO1 affect HCC progression are still rarely reported. Thus, this article was designed to investigate the potential molecular mechanism of miR-96 and FOXO1 affecting the progression of HCC to provide an important theoretical basis for HCC target treatment clinically.

## Methods

### Patients and samples

From January 2017 to October 2018, patients diagnosed with HCC for the first time in our hospital were included in the study. Patients with other serious organic diseases or pregnant or lactating women were excluded. A total of 60 patients were included. Tumor tissues as well as their corresponding paracancerous tissues (non-tumor tissues) were collected. This research had obtained informed consent from all patients. The approval of our hospital’s ethics committee was also obtained.

### Cell culture

Human normal hepatic cell line (L02) and hepatoma cell line (HepG2) were (purchased from Shanghai Institutes for Biological Sciences, Chinese Academy of Sciences) maintained in DMEM medium containing 10% fetal bovine serum (FBS) in a humidified incubator at 37 °C, 5% CO_2_.

### Cell transfection

HepG2 cells were prepared as cell suspension by serum-free DMEM at a density of 1 × 10^5^ cells/mL. They were inoculated in 6-well plates with 1 mL cell suspension per well. Transfection was performed on these cells by using miR-96 mimics and its negative control. These transfected cells were served as miR-mimics group and miR-NC group, respectively. FOXO1 expression vector and empty vector were obtained from Shanghai Jikai Gene Chemical Technology Co., Ltd., China. Co-transfection was performed on HepG2 cells by miR-96 negative control and empty vector (NC + Vector group), or by miR-96 mimics and empty vector (mimics + Vector group), or by miR-96 mimics and FOXO1 expression vector (mimics + FOXO1 group). All transfection was performed according to the Lipofectamine 2000 transfection kit (Thermo Fisher Scientific, Waltham, MA, USA) instructions. Residual serum-free DMEM in each well was replaced by DMEM containing 10% FBS after 6 h incubation. Cells of each group were continuously subjected to 72 h incubation. Under an inverted fluorescence microscopy, we observed that more than 90% of cells in each group showed green fluorescent protein expression. Cells with strong fluorescence intensity were screened and further cultured to obtain pure successfully infected cells. In this study, HepG2 cells without any treatment were used as Blank group. All cells were placed the humidified incubator at 37 °C, 5% CO_2_.

### Luciferase reporter assay

Based on the binding sites of FOXO1 and miR-96 predicted by Target Scan, mutant-type (MT) and wild-type (WT) sequences of FOXO1 were separately designed. These sequence fragments were cloned and integrated into vectors. HepG2 cells were co-transfected by using recombinant plasmid of MT FOXO1 sequences and miR-96 mimics (MT + mimics group), or by using recombinant plasmid of MT FOXO1 sequences and miR-96 negative control (MT + NC group), or by using recombinant plasmid of WT FOXO1 sequences and miR-96 mimics (WT + mimics group), or by using recombinant plasmid of WT FOXO1 sequences and miR-96 negative control (WT + NC group). After 48 h incubation in the humidified incubator at 37 °C, 5% CO_2_, luciferase assay was performed using the Dual-Luciferase Reporter assay kit (Promega, USA).

### Cell proliferation assay

Cells of NC + Vector group, mimics + Vector group and mimics + FOXO1 group were seeded in 96-well plates at a density of 1 × 10^3^ cells per well. They were maintained in the humidified incubator at 37 °C, 5% CO_2_ for 24, 48 and 72 h. MTT solution (5 mg/mL) was added with 20 μL per well for 4 h incubation at 37 °C. Residual liquid in each well was then replaced by 150 μL dimethyl sulfoxide (DMSO). Lightly shaking for 10 min was necessary to promote complete dissolution of formazan crystals. The absorbance of each well was measured at a wavelength of 495 nm (OD495 value) by an enzyme-linked immunosorbent assay.

### Cell migration and invasion assay

Migration and invasion of cells in NC + Vector group, mimics + Vector group and mimics + FOXO1 group were examined using Transwell assay. Briefly, cells of each group were prepared as cell suspension by serum-free DMEM at a density of 1 × 10^3^ cells/mL. Transwell chambers were placed in 24-well plates. A total of 1 mL cell suspension was added into the upper champer of the Transwell chamber (with or without Matrigel). In addition, 1 mL DMEM (10% FBS) was also added into the bottom of each well. All plates were placed in the humidified incubator at 37 °C, 5% CO_2_ for 24 h incubation. Residual medium in the upper champer was removed. The penetrated cells in the lower chamber were fixed with formaldehyde for 5 min, followed by 10 min staining with crystal violet. Cells were observed under an inverted microscope and 5 fields were randomly selected for cell counting.

### In vivo study by nude mice

Cell suspension (200 μL, 1 × 10^7^ cells/mL) of NC + Vector group, mimics + Vector group and mimics + FOXO1 group were injected subcutaneously into nude mice, respectively. Cell suspension of each group was injected with 10 nude mice (4–5 weeks, Tianjin Purcell Biotechnology Co., Ltd., China). All nude mice were sacrificed 5 weeks after injection to obtain subcutaneous tumor tissues. The long (a) and short (b) diameter of tumor tissues was measured to calculate tumor volume according to the following formula: tumor volume = (a × b^2^)/2. Tumor tissues were fixed with 10% formaldehyde and embedded in paraffin for subsequent immunohistochemical detection. In addition, the expression of AKT, GSK-3β, β-catenin, p-AKT, p-GSK-3β and p-β-catenin in tissues were calculated by western blot.

### Immunohistochemical detection

Paraffin-embedded tumor tissues were sequentially subjected to slicing and each tissue was cut into 10 consecutive sections. After all tissues were dewaxed, hydrated and antigen repaired, H_2_O_2_ (3%) was used to eliminate endogenous peroxidase activity for 10 min, followed by 10 min blocking with goat serum. FOXO1 antibody and biotinylated secondary antibody were used to incubate tissue sections. DAB chromogenic reaction, hematoxylin counterstaining and alcohol dehydration was then carried out. After sealed with neutral gel, tissue sections were viewed under a microscope and 5 fields of each section was randomly selected to count FOXO1 positive cells. Cells with brown particles in the cytoplasm were considered as FOXO1 positive cells.

### Quantitative real time polymerase chain reaction (qRT-PCR)

Total RNA in tumor tissues and cells was obtained by Trizol kit (Thermo Fisher Scientific, Waltham, MA, USA) according to the the instruction manual. The corresponding single-stranded cDNA template was obtained by reverse transcription.

PCR reaction was performed according to the following reaction procedure: 95 °C for 20 s, 58 °C for 30 s and 74 °C for 30 s, with 40 cycles. The primer sequences involved in this study were as follows: miR-96, forward, GCCCGCTTTGGCACTAGCACATT, reverse, GTGCAGGGTCCGAGGT; U6, forward, GCTTCGGCAGCACATATACTAAAA, reverse, CGCTTCACGAATTTGCGTGTCAT; FOXO1, forward, AGGGTTAGTGAGCAGGTTACAC, reverse, TGCTGCCAAGTCTGACGAAA; GAPDH, forward, GTCGATGGCTAGTCGTAGCATCGAT, reverse, TGCTAGCTGGCATGCCCGATCGATC. Data was processed by 2^−ΔΔCt^ method.

### Western blot analysis

RIPA cell lysis buffer was used to extract total proteins in tissues and cells. In addition, nucleus and cytoplasm of cells in each group was also separated by using Cytoplasmic & Nuclear RNA Purification Kit (Pumai Biotechnology Co., Ltd., Shanghai, China). Proteins concentration of each sample was determined using BCA kit (Thermo Fisher Scientific, Waltham, MA, USA). Sodium dodecyl sulfate polyacrylamide gel electrophoresis (SDS-PAGE) at 120 V was performed on these proteins for 1–2 h. Proteins were transferred onto PVDF membrane for 2 h blocking with 5% skimmed milk for 2 h, followed by incubation with primary antibodies (mouse anti-rabbit FOXO1, AKT, GSK-3β, β-catenin, p-AKT, p-GSK-3β and p-β-catenin antibodies, 1:1000, Cell Signaling Technology) for 12 h at 4 °C. After being rinsed 3 times with TBST, the membrane was incubated with goat anti-mouse IgG secondary antibodies (Beijing Zhongshan Jinqiao Biotechnology Co., Ltd., China) for 1 h at room temperature. Three times rinsing with TBST were also performed. Immunoblots were visualized using an enhanced chemiluminescence detection system (Amersham Pharmacia Biotech, Uppsala, Sweden). In this research, GAPDH was served as internal reference for the expression of total protein or cytoplasmic protein, while Histone 3 was considered internal reference for nuclear protein expression.

### Immunofluorescence

Cells of NC + Vector group, mimics + Vector group and mimics + FOXO1 group were inoculated in 6-well plates (with coverslip) for 12 h incubation in the incubator at 37 °C, 5% CO_2_. Pre-cooled anhydrous methanol at − 20 °C was used to fix cells for 15 min at 4 °C. After rinsed with PBS containing 5% Triton X-100 2 times, cells were blocked with 10% serum for 20 min, followed by 2 h incubation with rabbit anti-human β-catenin antibody as well as 1 h incubation with FITC-labeled goat anti-rabbit fluorophoric secondary antibody. After rinsed with PBS, the nuclei were stained with DAPI for 1 h. PBS rinsing was performed again and cells were observed under a fluorescence microscope.

### Statistical analysis

The t-test was used for comparison between two groups, and one-way analysis of variance test was used for comparison among three or more groups. Data was processed by SPSS 17.0. Each experiment was repeated three times. *P* < 0.05 was regarded as statistically significant.

## Results

### Up-regulation of miR-96 and down-regulation of FOXO1 in tumor tissues and HepG2 cells

**Fig. 1:**
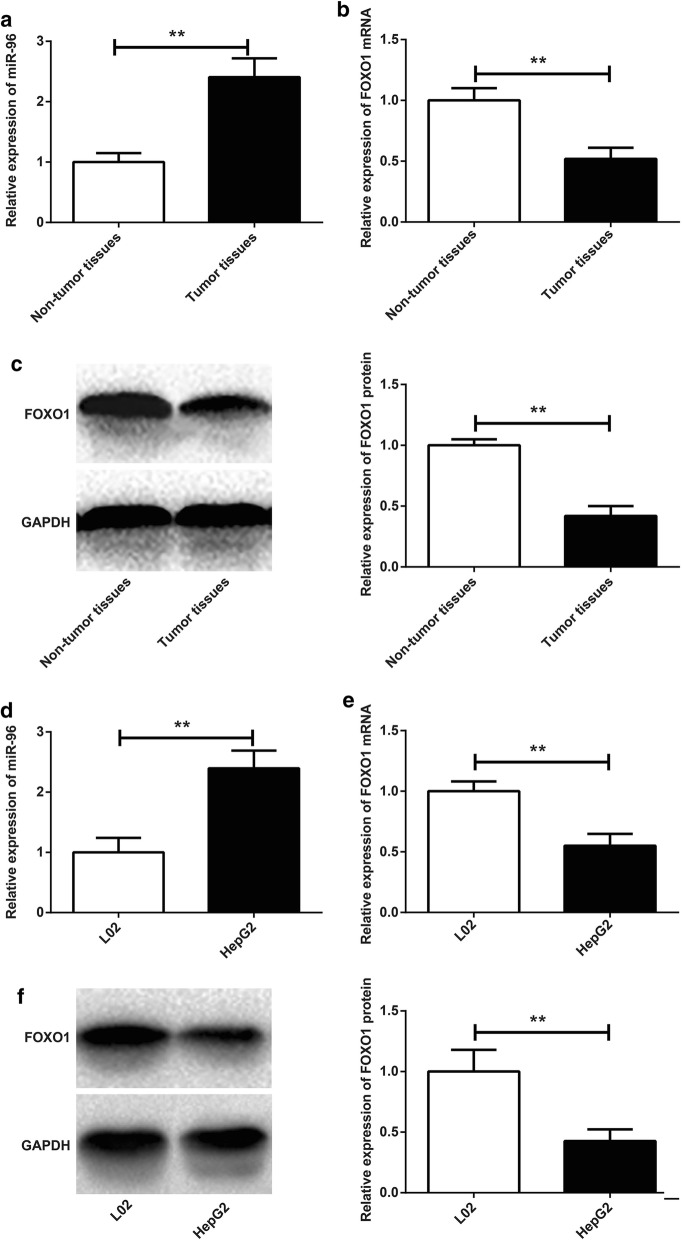
Up-regulation of miR-96 and down-regulation of FOXO1 in tumor tissues and HepG2 cells. **a** miR-96 relative expression in tumor tissues and non-tumor tissues by qRT-PCR; **b** FOXO1 mRNA relative expression in tumor tissues and non-tumor tissues by qRT-PCR; **c** FOXO1 protein relative expression in tumor tissues and non-tumor tissues by Western blot; **d** miR-96 relative expression in L02 and HepG2 cells by qRT-PCR; **e** FOXO1 mRNA relative expression in L02 and HepG2 cells by qRT-PCR; **f** FOXO1 protein relative expression in L02 and HepG2 cells by Western blot. **P < 0.01

miR-96 relative expression in tumor tissues was significantly higher than that in non-tumor tissues (*P* < 0.01) (Fig. 1a), whlie FOXO1 mRNA and protein relative expression was significantly lower in tumor tissues than that in non-tumor tissues (*P* < 0.01) (Fig. 1b, c). We also explored miR-96 and FOXO1 expression in L02 and HepG2 cells. Significantly increased miR-96 relative expression and dramatically decreased FOXO1 mRNA and protein relative expression was found in HepG2 cells than those in L02 cells (*P* < 0.01) (Fig. 1d–f). Furthermore, the effect of miR-96 and FOXO1 protein expression level on HCC patients’ prognosis was also explored. Lymph node metastasis, infiltration and differentiation were three important indicators of HCC patients’ prognosis. As shown in Table 1, high miR-96 expression and low FOXO1 protein expression was dramatically associated with positive lymph node metastasis and infiltration, and low-medium differentiation (*P* < 0.05), suggesting that miR-96 up-regulation and FOXO1 down-regulation predicted poor prognosis in HCC patients.

**Table 1:** The relationship between clinical features and expression levels of miR-96 and FOXO1

Clinical features	n	Relative of miR-96 expression	*t* value	*P* value	Relative of FOXO1 expression	*t* value	*P* value
Lymph node metastasis			2.61	0.02		− 2.14	0.04
Positive	11	2.45 ± 0.29			0.31 ± 0.13		
Negative	16	2.24 ± 0.12			0.44 ± 0.17		
Infiltration			2.34	0.03		− 2.55	0.02
Positive	13	2.53 ± 0.26			0.38 ± 0.11		
Negative	14	2.33 ± 0.18			0.51 ± 0.15		
Differentiation			2.15	0.04		− 2.16	0.04
Low-medium	19	2.38 ± 0.22			0.35 ± 0.12		
High	8	2.19 ± 0.18			0.48 ± 0.19		

### FOXO1 was directly suppressed by miR-96

**Fig. 2:**
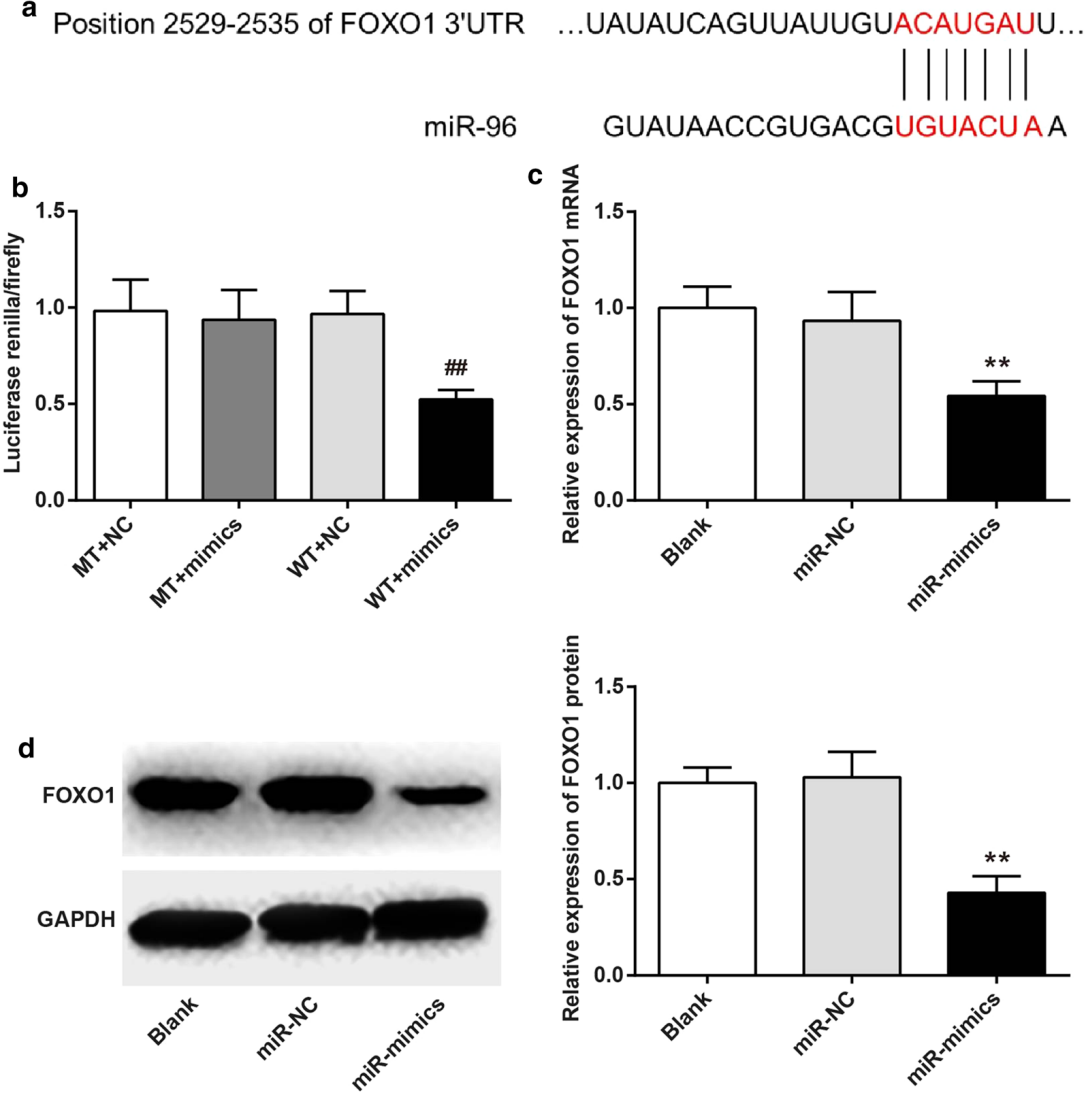
FOXO1 was directly suppressed by miR-96. **a** Prediction of binding sites for FOXO1 and miR-96 by Target Scan; **b** Luciferase reporter assay; **c** FOXO1 mRNA expression by qRT-PCR; **d** FOXO1 protein expression by Western blot. ^##^*P* < 0.01 when compared with WT + NC group; **P < 0.01 when compared with Blank group or miR-NC group

By Target Scan, we speculated that FOXO1 might be a potential downstream target of miR-96 (Fig. 2a). To verify the above hypothesis, luciferase reporter assay was carried out. The results showed that no significant difference in the intensity of luciferase activity was found between MT + NC group and MT + mimics group. However, the intensity of luciferase activity of WT + mimics group was significantly lower than that of WT + NC group (*P* < 0.01) (Fig. 2b). The regulatory of miR-96 on FOXO1 expression was further verified through transfection. Compared with Blank group and miR-NC group, the relative expression of FOXO1 mRNA and protein in miR-mimics group was significantly decreased (*P* < 0.01) (Fig. 2c, d). Based on these results, it might be concluded that FOXO1 was directly suppressed by miR-96.

### FOXO1 inhibited proliferation, migration and invasion of HepG2 cells induced by miR-96 in vitro

**Fig. 3:**
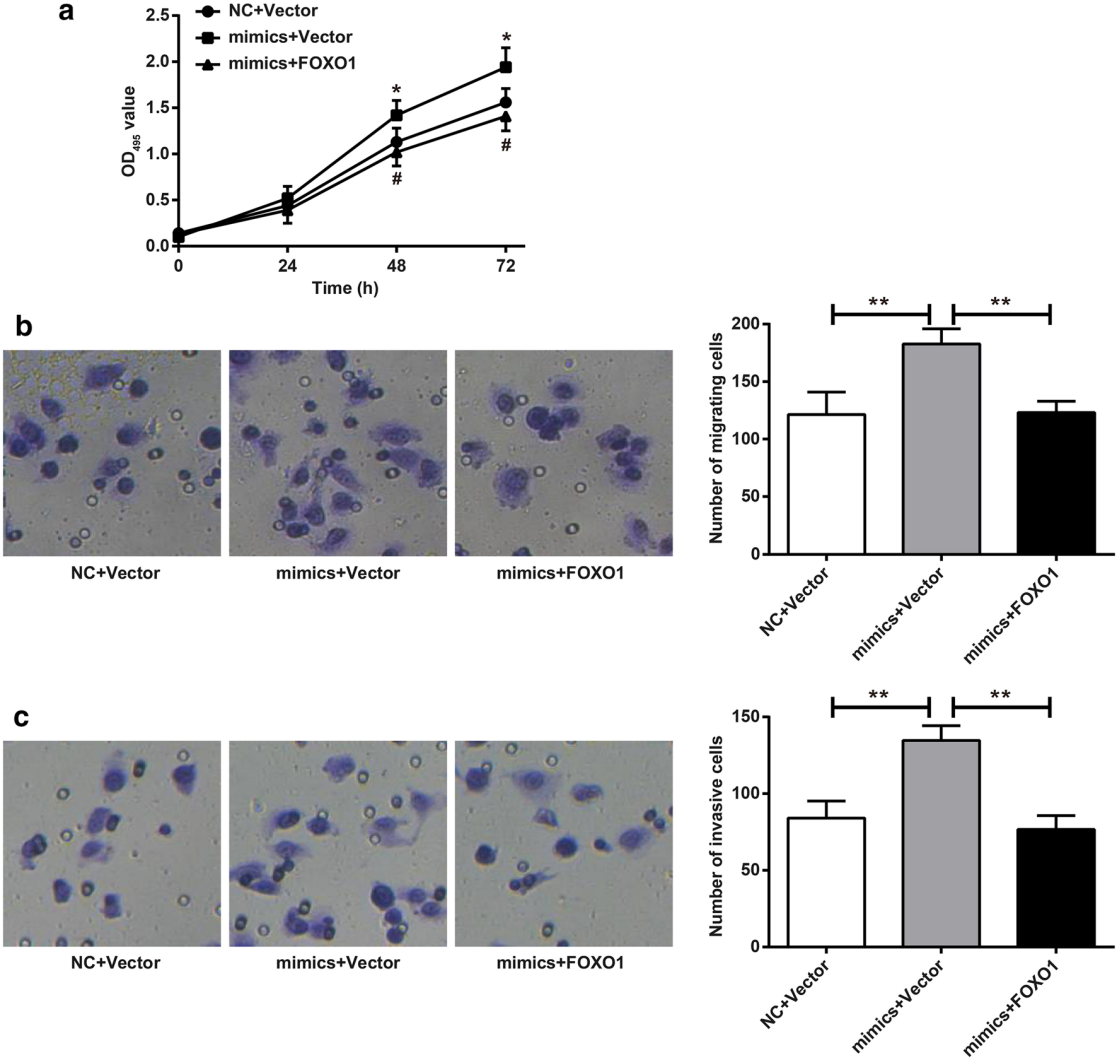
FOXO1 inhibited proliferation, migration and invasion of HepG2 cells induced by miR-96 in vitro. **a** Detection of cells proliferation by MTT assay. *P < 0.05 when compared with NC + Vector group, ^#^*P* < 0.05 when compared with mimics + Vector group; **b** Detection of cells migration by Transwell. **P < 0.01; **c** Detection of cells invasion by Transwell. **P < 0.01

Compared with NC + Vector group, the OD495 value of mimics + Vector group was significantly increased at 48 h and 72 h (*P* < 0.05), while at the same time, the OD495 value of mimics + FOXO1 group was significantly lower than that of mimics + Vector group (*P* < 0.05) (Fig. 3a). The number of migrating and invasive cells of mimics + Vector group was 183 ± 10 and 135 ± 8 respectively, which was significantly higher than that of NC + Vector group (121 ± 16, 84 ± 9, respectively) (*P* < 0.01). When compared with mimics + Vector group, significantly reduced migrating (123 ± 8) and invasive (75 ± 7) cells was observed in mimics + FOXO1 group (*P* < 0.01) (Fig. 3b, c). FOXO1 inhibited proliferation, migration and invasion of HepG2 cells induced by miR-96 in vitro.

### FOXO1 inhibited tumor growth induced by miR-96 in nude mice

**Fig. 4:**
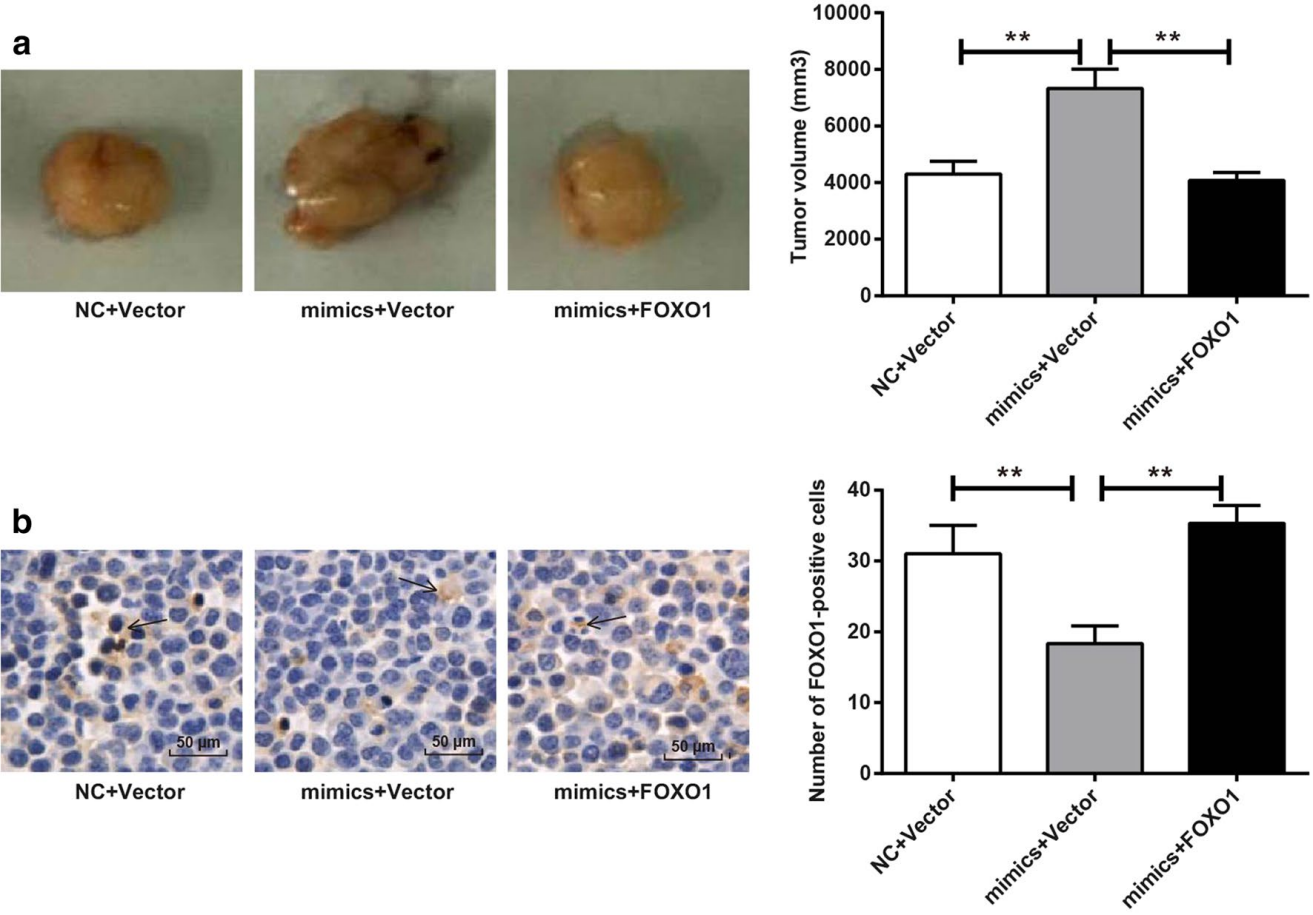
FOXO1 inhibited tumor growth induced by miR-96 in nude mice. **a** Tumor volume after subcutaneous transplantation for 5 weeks; **b** FOXO1-positive cells of xenograft tumors by immunohistochemical analysis. Arrow indicated FOXO1-positive cells. **P < 0.01

Five weeks after transplantation, tumor volume of mimics + Vector group was significantly higher than that of NC + Vector group (*P* < 0.01), and mimics + FOXO1 group had much smaller tumor volume when compared with mimics + Vector group (*P* < 0.01) (Fig. 4a). Immunohistochemical analysis of tumor tissues showed that significantly decreased FOXO1-positive cell numbers was found in mimics + Vector group than that in NC + Vector group, while significantly increased FOXO1-positive cell numbers was observed in mimics + FOXO1 group than that in mimics + Vector group (*P* < 0.01) (Fig. 4b). The above results indicated that FOXO1 inhibited tumor growth induced by miR-96 in nude mice.

### miR-96 activated AKT/GSK-3β/β-catenin signaling pathway by inhibition of FOXO1

**Fig. 5:**
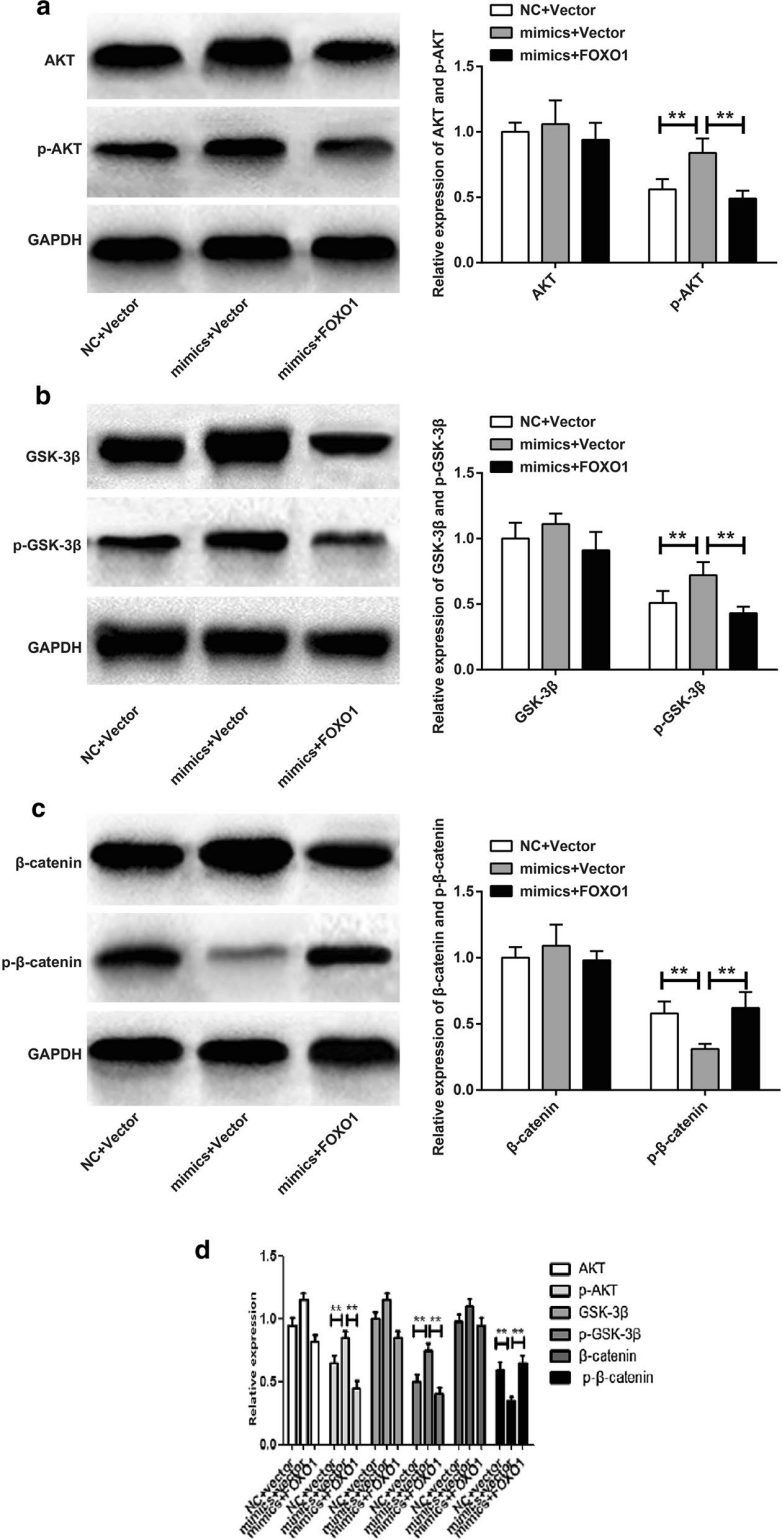
miR-96 activated AKT/GSK-3β/β-catenin signaling pathway through targeted inhibition of FOXO1. Detection of AKT and p-AKT protein (**a**), GSK-3 and p-GSK-3 protein (**b**), and β-catenin and p-β-catenin protein (**c**) by Western blot (**d**) by western blot in vivo. **P < 0.01

AKT/GSK-3β/β-catenin signaling pathway was an important tumor regulatory signaling pathway. In this article, we investigated the effect of miR-96 on AKT/GSK-3β/β-catenin signaling pathway. It could be noticed that there was no significant difference in AKT, GSK-3β, and β-catenin proteins relative expression among NC + Vector group, mimics + Vector group and mimics + FOXO1 group. However, significantly higher p-AKT, p-GSK-3β and significantly lower p-β-catenin was observed in mimics + Vector group than that in NC + Vector group (*P* < 0.01). Meanwhile, compared to mimics + Vector group, significantly decreased p-AKT, p-GSK-3β and significantly increased p-β-catenin was occurred in mimics + Vector group (*P* < 0.01) (Fig. 5a–c). The results of western blot showed that similarly results were also obtained in vivo (Fig. 5d). miR-96 activated AKT/GSK-3β/β-catenin signaling pathway through inhibition of FOXO1.

### FOXO1 inhibited β-catenin entry into the nucleus induced by miR-96

**Fig. 6:**
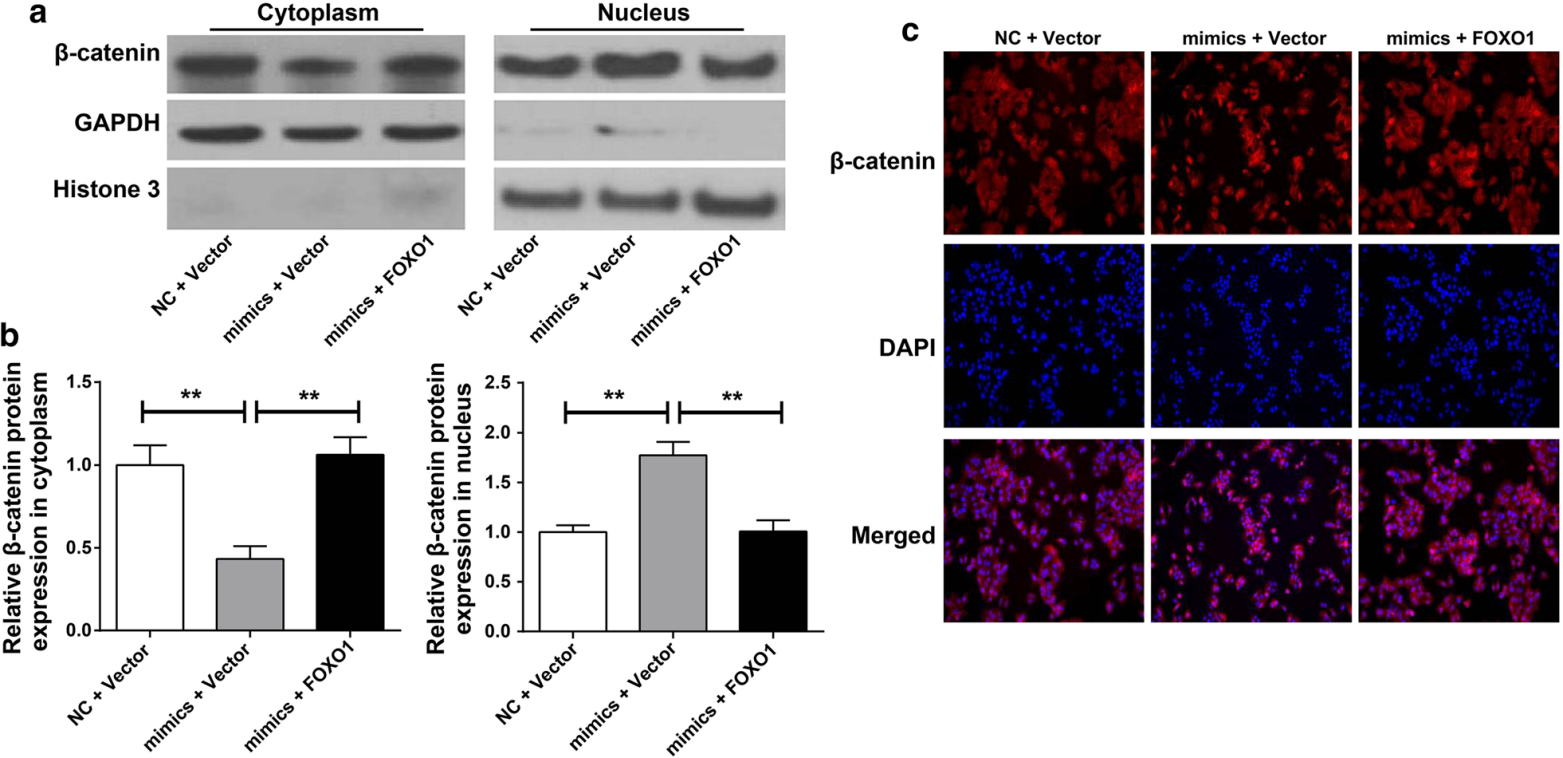
FOXO1 inhibited β-catenin entry into the nucleus induced by miR-96. Expression of β-catenin in nucleus (**a**) and cytoplasm (**b**) was detected by Western blot. **c** Distribution of β-catenin in nucleus and cytoplasm was monitored by immunofluorescence

Nucleus and cytoplasm of cells in each group was separated, and β-catenin protein expression in nucleus and cytoplasm was monitored by Western blot. As a result, we noticed that, compared with NC + Vector group, cells of mimics + Vector group had much higher nuclear β-catenin protein relative expression and obviously lower cytoplasmic β-catenin protein relative expression (*P* < 0.01). However, significantly lower nuclear β-catenin protein relative expression and markedly higher cytoplasmic β-catenin protein relative expression was found in cells of mimics + FOXO1 group when compared with mimics + Vector group (*P* < 0.01) (Fig. 6a, b). In addition, immunofluorescence results showed that, the main expression of β-catenin in NC + Vector group was located in the cytoplasm, while it was concentrated in the nucleus in mimics + Vector group. More importantly, we realized that, compared with mimics + Vector group, β-catenin protein in the nucleus was obviously reduced in mimics + FOXO1 group. Meanwhile, much increased β-catenin in the cytoplasm was found in mimics + FOXO1 group when compared with that in mimics + Vector group (Fig. 6c). These results suggested that FOXO1 inhibited β-catenin entry into the nucleus induced by miR-96.

## Discussion

We studied the expression of miR-96 in HCC and its mechanism of promoting HCC progression in this article. The results confirmed previous studies that miR-96 was up-regulated in HCC tissues and HepG2 cells. But more importantly, we noticed that miR-96 exerted carcinogenic effect by activating AKT/GSK-3β/β-catenin signaling pathway through targeting inhibition of FOXO1 in HepG2 cells.

Through in vitro studies, we observed that FOXO1 inhibited proliferation, migration and invasion of HepG2 cells induced by miR-96. In vivo studies also indicated that, FOXO1 inhibited tumor growth induced by miR-96 in nude mice and miR-96 promoted HCC progression through direct targeted inhibition of FOXO1. As we know, FOXO1 was a member of the FOXO family, which was widely distributed in human tissues and organs, such as heart, liver, prostate, peripheral blood, etc. [17–20]. FOXO1 was an important tumor suppressor gene and was down-regulated in many types of tumors [21]. It was involved in the proliferation, migration and invasion of a variety of tumor cells, including HCC cells. Previous literature indicated that FOXO1 was weakly expressed in HCC tissues, which resulted in severe malignant proliferation [22]. Yang et al. [18] also observed in their study that HCC cells proliferation was significantly promoted by inhibition of FOXO1. FOXO1 was regulated by multiple miRNAs, such as miR-3188, miR-411, miR-132, miR-145 and so on [5, 23–25]. In this study, we demonstrated that FOXO1 was a downstream target gene of miR-96 and miR-96 promoted HepG2 cells proliferation, migration and invasion by damaging the expression of FOXO1. This mechanism was proposed for the first time in HCC.

Equally important was that this research further illustrated that miR-96 activated AKT/GSK-3β/β-catenin signaling pathway by inhibition of FOXO1. Overexpression of miR-96 did not affect AKT, GSK-3β and β-catenin protein levels. However, it significantly increased p-AKT and p-GSK-3β protein levels and dramatically decreased p-β-catenin expression. Up-regulation of FOXO1 could weak this effect of miR-96. It could also be noted that miR-96 promoted β-catenin entry into the nucleus, while FOXO1 could inhibit the entry of β-catenin into the nucleus. As one of the most important tumor-related signaling pathways, the activation of AKT/GSK-3β/β-catenin signaling pathway played a pivotal role in the development of tumors. Akt was a serine/threonine protein kinase that played an important role in cell proliferation, differentiation, inhibition and apoptosis [26, 27]. In the AKT/GSK-3β/β-catenin signaling pathway, GSK-3β acts as a key gene in phosphorylation and degradation of β-catenin [28]. The activity of GSK-3β can be regulated by AKT phosphorylation at Ser9 [29, 30]. Phosphorylation of AKT promoted the phosphorylation of GSK-3β, which impaired the ability of GSK-3β to degrade β-catenin [31, 32]. β-catenin was the key molecule in this signaling pathway. After degradation of β-catenin was damaged, it would accumulate in the cytoplasm and eventually translocate into the nucleus. In the nucleus, β-catenin further activated its downstream genes to promote the progression of tumors [33, 34]. Therefore, preventing the activation of the AKT/GSK-3β/β-catenin signaling pathway and the entry of β-catenin into the nucleus could alleviate the development of tumors. As early as 2012, Fendler et al. [35] illustrated that FOXO1 was target gene of miR-96, and miR-96 could promote prostate cancer progression by directly inhibiting FOXO1 expression. In this study, we not only verified the above conclusion, but further found that in HCC, miR-96 might activated AKT/GSK-3β/β-catenin signaling pathway and promoted the entry of β-catenin into the nucleus by directly inhibition of FOXO1. This article firstly reported that miR96/FOXO1 was linked with AKT/GSK-3β/β-catenin signal pathway in HCC. This was an important innovation point of our research.

There were some limitations in this study. First, because of the limitation of experimental conditions, cell populations have not been sorted. Second, the expression of downstream genes of β-catenin could not be explored because of limited laboratory conditions. It would be be the focus of our future research. Finally, this paper first proposed that miR96/FOXO1 was linked with AKT/GSK-3β/β-catenin signal pathway in HCC. Of course, more researches still need to be carried out to confirm this finding, which will be the foucus of our future studies.

## Conclusion

To sum up, this article revealed that miR-96 was an oncogene in HCC and it was up-regulated in HCC tissues and HepG2 cells. Related mechanism was that miR-96 activated AKT/GSK-3β/β-catenin signaling pathway and further promoted the entry of β-catenin into the nucleus by targeted inhibition of FOXO1. This study provided a potential target for the molecular treatment of HCC, which had important theoretical value and clinical significance.
